# Comparative electrocardiographic analysis of midventricular and typical takotsubo syndrome

**DOI:** 10.3389/fcvm.2023.1286975

**Published:** 2023-12-04

**Authors:** Mireia Padilla-Lopez, Albert Duran-Cambra, David Belmar-Cliville, Marc Soriano-Amores, Sabiñe Arakama-Goikoetxea, Montserrat Vila-Perales, Walter Bragagnini, Laura Rodríguez-Sotelo, Pedro Peña-Ortega, Jesús Sánchez-Vega, Jose Carreras-Mora, Alessandro Sionis

**Affiliations:** ^1^ Cardiology Department, Hospital de la Santa Creu I Sant Pau, IIB-SantPau, Universidad Autónoma de Barcelona, Barcelona, Spain; ^2^ Centro de Investigación Biomédica en Red Enfermedades Cardiovasculares (CIBER-CV), Madrid, Spain

**Keywords:** takotsubo syndrome, midventricular, atypical TTS, typical TTS, electrocardiographic

## Abstract

**Introduction:**

Takotsubo syndrome (TTS) encompasses distinct variants, with midventricular (MV) as the most common atypical subtype. While electrocardiogram (ECG) abnormalities are well documented in typical TTS, they are less explored in MV-TTS.

**Methods:**

A retrospective case-control study was conducted where ECGs were reviewed at three time points from symptom onset (within the first 12 h, at 48 h, and at 5–7 days) and compared between patients with typical TTS (*n* = 33) and those with MV-TTS (*n* = 27), as classified by ventriculography.

**Results:**

12-h ECG findings revealed that typical TTS featured ST-segment elevation through anterior leads V3–V6, with maximal deviation in V3 (0.98 ± 0.99 mm) and V4 (0.91 ± 0.91 mm), whereas MV-TTS featured ST-segment depression in inferior leads (−0.24 ± 0.57 mm in II, −0.30 ± 0.52 mm in III, and −0.32 ± 0.47 mm in aVF) and in precordial leads V4–V6. In 48-h ECG findings, the most significant change was T wave inversion, which was more widespread and deeper in typical TTS, with the most pronounced negative T wave depths, exceeding 3 mm, observed in leads V3–V5; in contrast, in MV-TTS, T wave inversion was evident in fewer leads and showed less depth, with the most pronounced negative T waves reaching 1 mm at most in leads I, aVL, and V2. While the QTc interval was prolonged in both groups at 48 h, this prolongation was more pronounced in typical TTS than in MV-TTS (523 ± 52 ms vs. 487 ± 66 ms; *p *= 0.029). In ECGs at 5–7 days, results essentially returned to baseline.

**Conclusion:**

Patients with MV-TTS exhibited a distinctive pattern of ECG abnormalities, marked by ST-segment depression in inferolateral leads, less profound and less extensive T wave inversion that mostly affected leads I, aVL and V2, and attenuated QT interval prolongation compared to typical TTS.

## Introduction

Takotsubo syndrome (TTS) is characterized by the appearance of acute and reversible regional wall motion abnormalities (RWMAs), mostly dyskinesia, affecting different segments of the left ventricle (1). Depending on the location of these RWMAs, different types of TTS have been identified. The first described form was the typical TTS, which RWMAs affect the apical segments, making it the most frequently observed presentation (2). Subsequently, patterns associated with different RWMA distributions have been identified as atypical variants of TTS (3, 4).

Midventricular (MV) TTS, characterized by MV dyskinesia/hypokinesia and basal and apical hypercontractility, is the most frequent atypical variant, accounting for 20%–40% of all cases of TTS (3, 5), and increasingly diagnosed in clinical practice (6). However, the fact that data on MV-TTS are scarce and sometimes wrongly extrapolated from typical TTS has motivated recent studies of atypical TTS types, most especially MV-TTS, reported to have a different clinical profile: patients tend to be younger, and have a better left ventricular ejection fraction (LVEF) and lower brain natriuretic peptide (BNP) and troponin levels (6–10). Importantly, several studies have reported that patients with MV-TTS also have a better prognosis than patients affected by typical TTS (6, 9, 11) with potentially important implications for the clinical and hospital management of the different types of TTS.

Since TTS clinical presentation is usually very similar to that for acute coronary syndrome, electrocardiogram (ECG) interpretation plays a crucial role in initial evaluation of these patients (12–16). ECG abnormalities described in TTS mainly include ST-segment elevation at first instance (generally in precordial leads), followed by subsequent T wave inversion and QT interval prolongation in the following hours/days (2, 17–19). However, these ECG abnormalities are generally described from the ECGs of patients with typical TTS.

We therefore investigated whether MV-TTS is characterized by a pattern of ECG abnormalities different from typical TTS. This could potentially enhance clinical suspicion and facilitate early diagnosis of the specific clinical features profiling this emerging subgroup of patients.

## Materials and methods

### Study population

We conducted a single-center retrospective case-control study. Between January 2013 and April 2023, patients diagnosed with TTS who were discharged from our center were retrospectively included in a database (*n *= 309). Inclusion criteria were as follows: (1) age ≥18 years; (2) a TTS diagnosis according to InterTAK criteria (5); (3) clearly identifiable and exact time of symptom onset; (4) at least one ECG in the first 48 h; and (5) available ventriculography. Exclusion criteria were patients with left bundle branch block, right bundle branch block, non-specific intraventricular conduction disorder, and pacemaker stimulation (excluded due to the potential impact on repolarization from the baseline, which could introduce confounding factors in results interpretation). To standardize the sample and avoid classification errors in ECG timing and the TTS type, the inclusion criteria specified including only patients with a clearly identifiable and exact time of symptom onset and with an available ventriculography, as echocardiogram sometimes has limitations in defining the RWMA (and so could misclassify patients) and cardiac magnetic resonance is generally not available in the acute disease phase.

The study complied with ethical guidelines and was approved by the ethics committee of our center.

### Study variables

#### Clinical variables

Clinical variables, retrieved from hospital records, were as follows: (1) demographic and previous clinical history including comorbidities; (2) case history on admission and in-hospital clinical course; (3) laboratory blood tests during hospitalization; and (4) echocardiogram on admission.

#### ECG

Available ECGs (electronic or paper medical records) for each patient were classified into three different time periods: ECG-1, ECG-2, and ECG-3, obtained within the first 12 h, at 48 h, and at 5–7 days, respectively, from symptom onset. ECG recordings were anonymized, digitalized, and analyzed using electronic calipers (Cardio-Calipers software, Iconico). ECGs were obtained at a paper speed of 25 mm/s with amplification of 10 mm/mV. Analyzed in each ECG were heart rhythm, heart rate, and PR segment. The ST-segment was measured at the J point, and the R wave and T wave magnitudes were measured relative to the isoelectric line, defined as the level of the preceding TP segment. Corrected QT intervals (QTc) were measured using Bazett's formula (QTc = QT/RR interval). ECG measurements were made twice by two independent cardiologists who underwent prior training in caliper use. The resulting mean values were compared and any difference greater than ±0.2 mm was discussed by all the investigators.

#### Ventriculography

**Figure 1:**
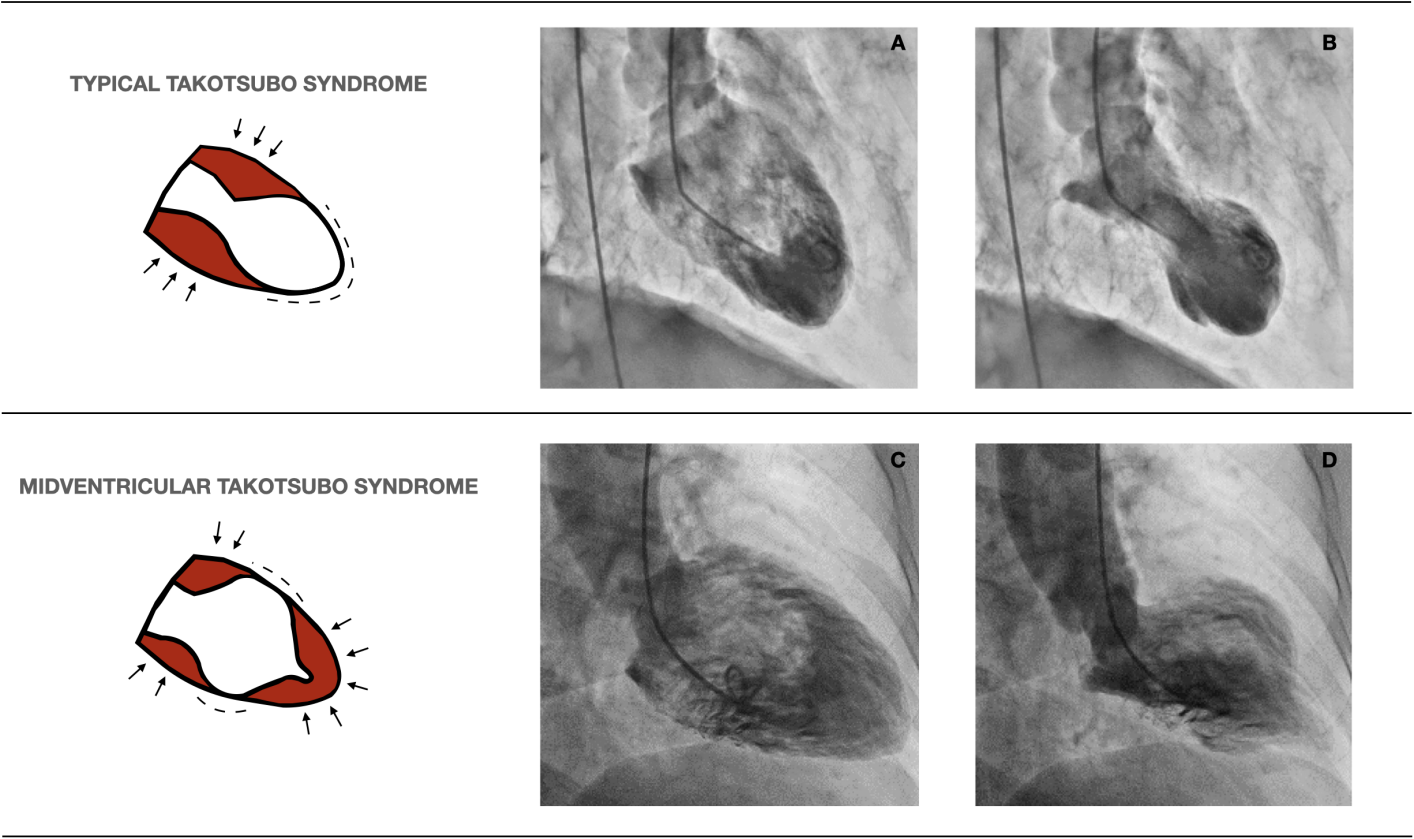
Left ventriculography images of representative cases of typical TTS and MV-TTS. The diagrams on the left illustrate normokinetic or hyperkinetic segments (arrows) and akinetic segments (dashes). (**A**) (end-diastole) and (**B**) (end-systole) exemplify typical TTS, while (**C**) (end-diastole) and (**D**) (end-systole) characterize MV-TTS. TTS, takotsubo syndrome; MV-TTS, midventricular TTS.

Left ventricular angiograms were performed in the 30° right anterior oblique projection, as is usual. Each ventriculography was reviewed to accurately classify TTS types. MV-TTS was characterized by akinesia of the middle portion of the left ventricular chamber, along with normokinesia or hyperkinesia of the apical and basal portions, while typical TTS exhibited akinesia of the middle-to-distal portion of the left ventricular chamber, and normokinesia or hyperkinesia of the basal portion. Figure 1 depicts left ventriculography images of representative typical TTS and MV-TTS cases.

### Statistical analysis

Categorical variables were described in terms of frequencies and percentages, and statistical differences were analyzed using the chi-square or Fisher exact test as appropriate. Continuous variables were described in terms of the mean and standard deviation (mean ± SD) or the median and interquartile range (IQR), and statistical differences were analyzed using the student-t test for independent normally distributed samples, or the Mann-Whitney test for non-normal distributions. A *p* value <0.05 was considered significant. All analyses were performed using Stata Statistical Software: Release 15.

## Results

**Figure 2:**
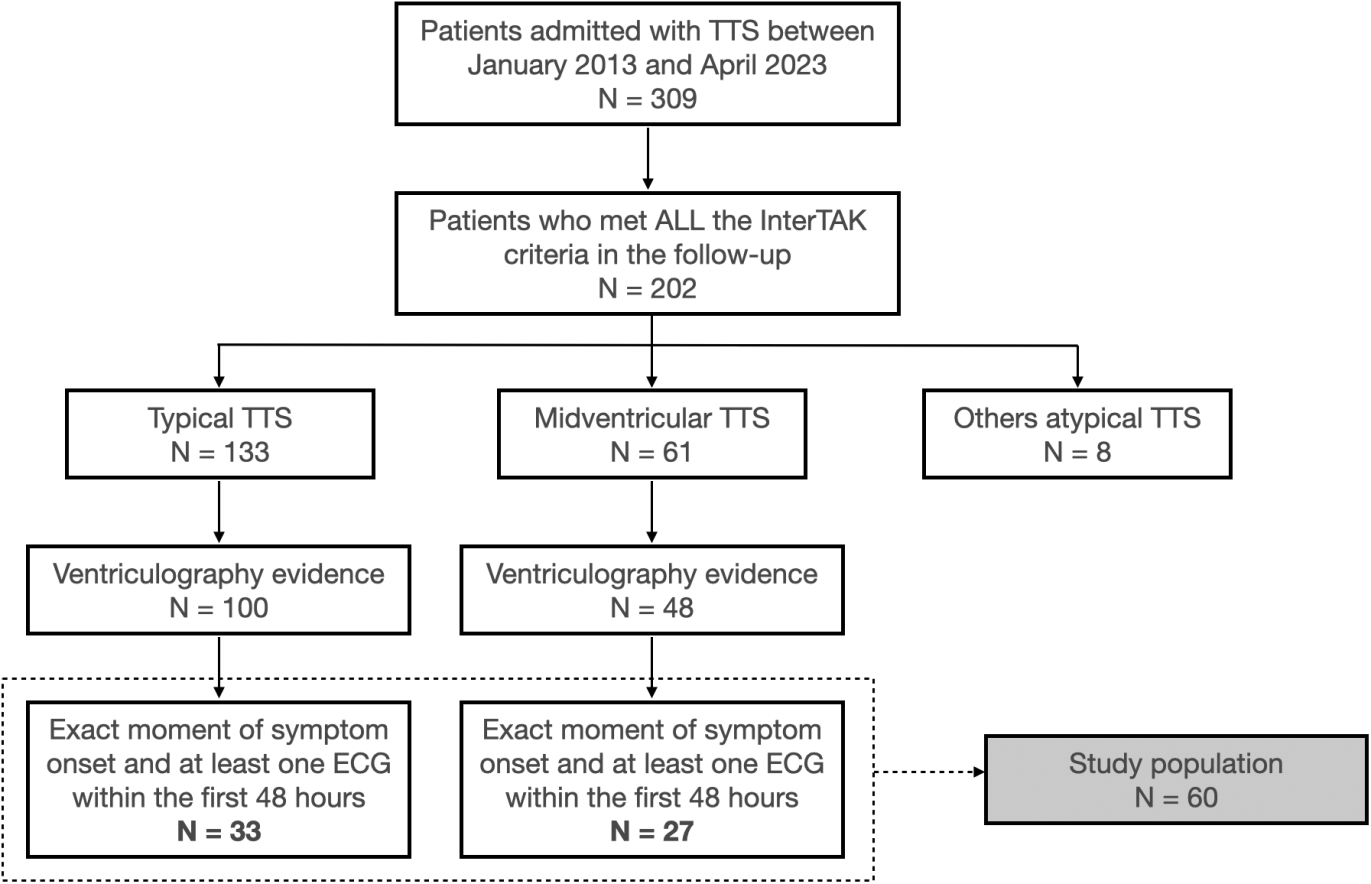
Study flowchart. ECG, electrocardiogram; TTS, takotsubo syndrome.

Between January 2013 and April 2023, 309 patients were admitted to our center with a TTS diagnosis, 60 of whom met the inclusion criteria and were entered in the study. After ventriculography review, 33 patients were classified as having typical TTS and 27 as MV-TTS, as depicted in Figure 2.

Table 1, summarizing baseline characteristics, reveals no differences in demographic characteristics, baseline disease, or laboratory parameters between the two groups. All patients were women, except for a single man with MV-TTS. Physical stressors were a more frequent trigger for MV-TTS (48%) than for typical TTS (24%), although this difference was not statistically significant. In both groups, chest pain followed by dyspnea were the most frequent initial symptoms. Regarding the echocardiographic findings on admission, the LVEF was similar in both groups, but moderate-to-severe mitral regurgitation (MR) and left ventricular outflow tract (LVOT) obstruction were significantly more prevalent in typical TTS than in MV-TTS (30% vs. 7% and 24% vs. 0%, respectively). Approximately a quarter of patients in each group presented with heart failure, and there were no in-hospital deaths.

**Table 1:** Characteristics of admitted patients overall and with typical TTS vs. MV-TTS.

	All patients (*n *= 60)	Typical TTS (*n *= 33)	MV-TTS (*n *= 27)	*p* value
Clinical variables				
Age, years	71 ± 11	71 ± 11	70 ± 10	0.710
Female	59 (98.3)	33 (100)	26 (96)	0.265
BMI, kg/m^2^	22 ± 4	22 ± 4	21 ± 3	0.961
Current smoker	12 (20)	7 (21)	5 (19)	0.795
Hypertension	33 (55)	21 (64)	12 (44)	0.137
Dyslipidemia	28 (47)	16 (48)	12 (44)	0.755
Diabetes mellitus	8 (13)	5 (15)	3 (11)	0.719
Known CAD	2 (3)	2 (6)	0	0.497
COPD	9 (15)	3 (9)	6 (22)	0.276
Psychiatric disorder	18 (30)	12 (36)	6 (22)	0.270
Neurological disorder	11 (18)	3 (9)	8 (30)	0.051
Trigger events				
Emotional stressor	22 (37)	15 (45)	7 (26)	0.131
Physical stressor	21 (35)	8 (24)	13 (48)	
No identifiable cause	17 (28)	10 (30)	7 (26)	
Initial symptoms				
Chest pain	40 (67)	22 (67)	18 (67)	0.930
Dyspnea	12 (20)	6 (18)	6 (22)	
Syncope	4 (7)	3 (9)	1 (4)	
Cardiac arrest	4 (7)	2 (6)	2 (7)	
Echocardiographic parameters				
LVEF (%)	43 ± 9	41 ± 9	45 ± 9	0.087
Moderate to severe MR	12 (20)	10 (30)	2 (7)	**0** **.** **049**
Significant LVOT obstruction	8 (13)	8 (24)	0	**0** **.** **006**
Laboratory parameters^a^				
Peak troponin T, ng/L^b^	579 [368–1,173]	633 [368–1,173]	566 [356–1,178]	0.766
Peak CK, U/L	190 [124–327]	186 [127–308]	193 [124–341]	0.849
Peak NT-proBNP, pg/ml	3,749 [2,330–6,682]	5,166 [2,408–9,369]	3,440 [2,330–4,473]	0.370
In-hospital complications				
Heart failure	16 (27)	9 (27)	7 (26)	0.907
Cardiogenic shock	5 (8)	3 (9)	2 (7)	1
CVA	1 (2)	0	1	0.450
Death	0	0	0	>0.999

Data are expressed as *n* (%) or mean ± standard deviation. BMI, body mass index; CAD, coronary artery disease; CK, creatine kinase; COPD, chronic obstructive pulmonary disease; CVA, cerebral vascular accident; ECG, electrocardiogram; LVEF, left ventricle ejection fraction; LVOT, left ventricle outflow tract; MR, mitral regurgitation; NT-proBNP, N-terminal pro-B-type natriuretic peptide.

^a^ Data are expressed as median [interquartile range].

^b^ High sensitivity cardiac troponin T. 99th percentile upper reference limit of 14 ng/L.

Values in bold are values with significant value considering *p* value.

In total, 139 ECGs were analyzed, 54 classified as ECG-1 (within 12 h from symptom onset), 53 as ECG-2 (48 h from symptom onset), and 32 as ECG-3 (5–7 days from symptom onset).

**Figure 3:**
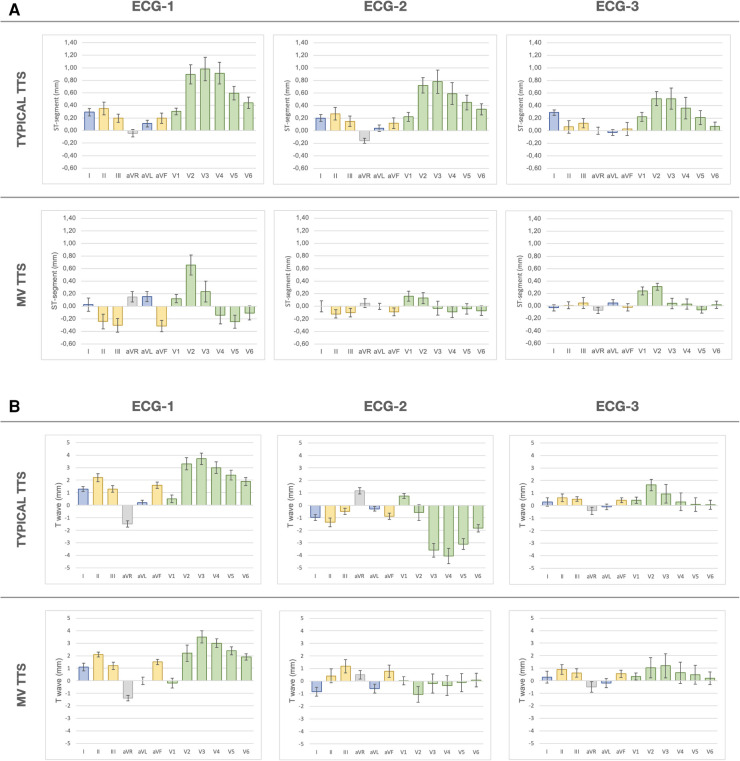
ECG analyses of ST-segment and T wave evolution (ECG-1, ECG-2, and ECG-3) for typical TTS and MV-TTS. The bars depict mean (standard error) values in mm across 12 conventional leads. (**A**) ST-segment evolution. (**B**) T wave evolution. ECG, electrocardiogram; ECG-1,12 h from symptom onset; ECG-2, 48 h from symptom onset; ECG-3, 5–7 days from symptom onset; TTS, takotsubo syndrome; MV-TTS, midventricular TTS.

Table 2 summarizes ECG characteristics and Figure 3 illustrates ST-segment and T wave changes in ECG-1, ECG-2, and ECG-3 for the typical TTS compared to the MV-TTS.

**Table 2:** ECG-1, ECG-2 and ECG-3 parameters for patients with typical TTS and MV-TTS.

	ECG-1			ECG-2			ECG-3		
	Typical TTS (*n *= 28)	MV-TTS (*n *= 26)	*p* value	Typical TTS (*n *= 29)	MV-TTS (*n *= 24)	*p* value	Typical TTS (*n *= 17)	MV-TTS (*n *= 15)	*p* value
Sinus rhythm, *n* (%)	26 (93)	25 (96)	1	28 (97)	23 (96)	1	16 (94)	14 (93)	1
Atrial fibrillation, *n* (%)	2 (7)	1 (4)	1	1 (3)	1 (4)	1	1 (6)	1 (7)	1
Heart rate, bpm	95 ± 23	84 ± 23	0.090	83 ± 21	75 ± 17	0.163	77 ± 19	64 ± 9	**0** **.** **026**
PR segment, ms	165 ± 21	169 ± 29	0.613	159 ± 20	162 ± 42	0.745	154 ± 20	154 ± 23	0.984
QTc, ms	444 ± 29	454 ± 30	0.219	523 ± 52	487 ± 66	**0** **.** **029**	472 ± 35	423 ± 33	**<0** **.** **01**
ST-segment, mm									
I	0.29 ± 0.32	0.03 ± 0.51	**0** **.** **026**	0.2 ± 0.28	0 ± 0.42	**0** **.** **04**	0.29 ± 0.17	−0.03 ± 0.18	0.332
II	0.35 ± 0.54	−0.24 ± 0.57	**<0** **.** **01**	0.27 ± 0.53	−0.12 ± 0.29	**<0** **.** **01**	0.06 ± 0.41	0.01 ± 0.21	0.622
III	0.20 ± 0.33	−0.30 ± 0.52	**<0** **.** **01**	0.15 ± 0.43	−0.1 ± 0.29	**0** **.** **032**	0.12 ± 0.30	0.05 ± 0.34	0.578
aVR	−0.43 ± 0.31	0.15 ± 0.42	0.062	−0.16 ± 0.22	0.05 ± 0.34	**0** **.** **012**	0 ± 0.23	−0.07 ± 0.19	0.390
aVL	0.11 ± 0.29	0.15 ± 0.39	0.664	0.04 ± 0.26	0 ± 0.23	0.487	−0.03 ± 0.18	0.05 ± 0.19	0.188
aVF	0.19 ± 0.43	−0.32 ± 0.47	**<0** **.** **01**	0.12 ± 0.46	−0.09 ± 0.28	0.070	0.03 ± 0.42	−0.02 ± 0.22	0.689
V1	0.30 ± 0.29	0.12 ± 0.32	**0** **.** **036**	0.22 ± 0.39	0.16 ± 0.37	0.597	0.22 ± 0.29	0.24 ± 0.24	0.916
V2	0.90 ± 0.82	0.65 ± 0.78	0.285	0.72 ± 0.66	0.13 ± 0.41	**<0** **.** **01**	0.51 ± 0.45	0.31 ± 0.21	0.144
V3	0.98 ± 0.99	0.23 ± 0.85	**<0** **.** **01**	0.78 ± 1	−0.03 ± 0.52	**<0** **.** **01**	0.51 ± 0.69	0.04 ± 0.34	**0** **.** **024**
V4	0.91 ± 0.91	−0.14 ± 0.71	**<0** **.** **01**	0.59 ± 0.94	−0.09 ± 0.42	**<0** **.** **01**	0.36 ± 0.71	0.03 ± 0.32	0.111
V5	0.60 ± 0.56	−0.25 ± 0.52	**<0** **.** **01**	0.45 ± 0.63	−0.04 ± 0.41	**<0** **.** **01**	0.21 ± 0.46	−0.06 ± 0.21	**0** **.** **044**
V6	0.44 ± 0.49	−0.11 ± 0.56	**<0** **.** **01**	0.34 ± 0.47	−0.07 ± 0.38	**<0** **.** **01**	0.07 ± 0.28	0.02 ± 0.22	0.582
T wave, mm									
I	1.3 ± 1	1.1 ± 1.5	0.632	−0.97 ± 1.29	−0.85 ± 1.74	0.773	0.29 ± 1.38	0.28 ± 1.80	0.979
II	2.2 ± 1.7	2.1 ± 1	0.800	−1.36 ± 1.82	0.41 ± 2.73	**<0** **.** **01**	0.62 ± 1.25	0.91 ± 1.51	0.566
III	1.3 ± 1.3	1.2 ± 1.4	0.671	−0.48 ± 1.29	1.18 ± 2.56	**<0** **.** **01**	0.52 ± 0.70	0.63 ± 1.26	0.758
aVR	−1.5 ± 1.3	−1.4 ± 1.1	0.747	1.16 ± 1.37	0.51 ± 1.66	0.123	−0.42 ± 1.24	−0.5 ± 1.61	0.871
aVL	0.2 ± 1	−0.01 ± 1.4	0.476	−0.28 ± 0.99	−0.60 ± 1.72	0.155	−0.10 ± 0.88	−0.18 ± 1.36	0.847
aVF	1.6 ± 1.4	1.5 ± 1	0.821	−0.88 ± 1.38	0.79 ± 2.41	**<0** **.** **01**	0.43 ± 0.82	0.55 ± 1.10	0.718
V1	0.5 ± 1.6	−0.2 ± 1.9	0.160	0.75 ± 1.02	0.03 ± 1.59	0.050	0.42 ± 1.01	0.33 ± 1.08	0.821
V2	3.3 ± 2.6	2.2 ± 3.2	0.182	−0.57 ± 3.39	−1.06 ± 3.06	0.587	1.64 ± 1.87	1.05 ± 3.13	0.514
V3	3.7 ± 2.4	3.5 ± 2.5	0.692	−3.59 ± 2.93	−0.2 ± 3.73	**<0** **.** **01**	0.94 ± 3.09	1.19 ± 3.67	0.839
V4	3 ± 2.5	3 ± 1.8	0.996	−4.06 ± 3.30	−0.36 ± 3.84	**<0** **.** **01**	0.29 ± 2.94	0.64 ± 3.31	0.756
V5	2.4 ± 2.1	2.4 ± 1.6	0.971	−3.10 ± 2.38	−0.12 ± 3.47	**<0** **.** **01**	0.08 ± 2.27	0.47 ± 2.90	0.678
V6	1.9 ± 1.6	1.9 ± 1.3	0.926	−1.84 ± 1.62	0.08 ± 2.68	**<0** **.** **01**	0.06 ± 1.50	0.21 ± 1.96	0.810

All values are expressed as mean ± standard deviation except where otherwise indicated. ECG, Electrocardiogram; ECG-1, 12 h from symptom onset; ECG-2, 48 h from symptom onset; ECG-3, 5–7 days from symptom onset; TTS, takotsubo syndrome; MV-TTS, midventricular TTS.

Bold values denote statistical significance (*p* < 0.05).

In ECG-1, significant differences were observed in the ST-segment deviation between the two groups. Patients with typical TTS presented ST-segment elevation in lead I extending throughout anterior leads V3–V6, with maximal deviation in leads V3 (0.98 ± 0.99 mm) and V4 (0.91 ± 0.91 mm). In contrast, patients with MV-TTS presented ST-segment depression in inferior leads (−0.24 ± 0.57 mm in II, −0.30 ± 0.52 mm in III, and −0.32 ± 0.47 mm in aVF) extending throughout precordial anterolateral leads V4 (−0.14 ± 0.71 mm), V5 (−0.25 ± 0.52 mm), and V6 (−0.11 ± 0.56 mm). No differences were observed regarding T wave and QT interval analysis. T waves did not exhibit inversion, and the QTc interval was in the upper reference range of normality.

**Figure 4:**
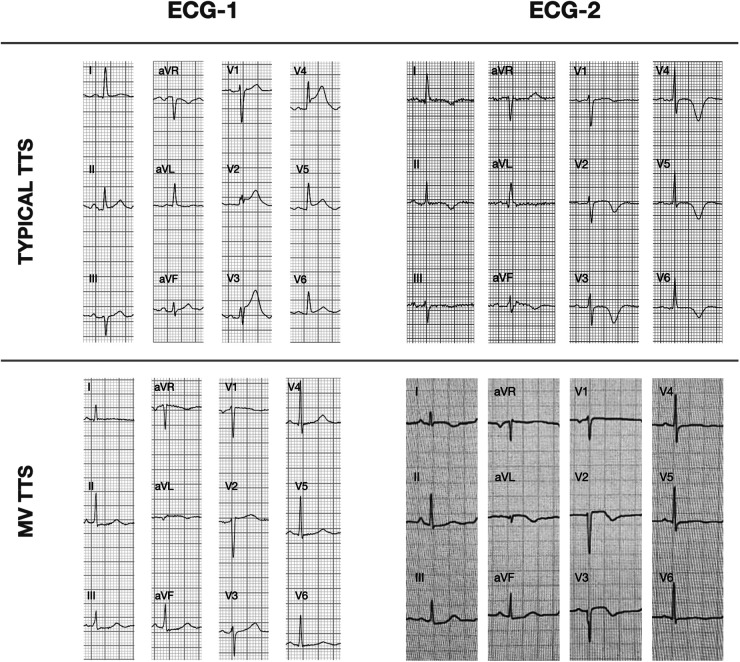
Representative ECG-1 and ECG-2 findings for typical TTS and MV-TTS. ECG, electrocardiogram; ECG-1,12 h from symptom onset; ECG-2, 48 h from symptom onset; TTS, takotsubo syndrome; MV-TTS, midventricular TTS.

In ECG-2, the ST-segment remained elevated in leads V2–V6 in typical TTS, while ST-segment depression persisted in inferior leads and from V4–V6 in MV-TTS; all those ST-segment deviations were less pronounced with respect to the isoelectric line than in ECG-1. The main ECG-2 changes were observed in T waves and QT intervals. In typical TTS, significant T wave inversions were evident in all leads except V1, and the most pronounced negative T waves were observed in leads V3 (−3.59 ± 2.93 mm), V4 (−4.06 ± 3.30 mm), and V5 (−3.10 ± 2.38 mm). In contrast, in MV-TTS fewer leads displayed T wave inversion and depth was less, with the most pronounced negative T waves occurring in I (−0.85 ± 1.74 mm), aVL (−0.60 ± 1.72 mm), and V2 (−1.06 ± 3.06 mm). In both groups, the QTc interval was prolonged, with mean values exceeding the upper limit of normal. Notably, in typical TTS QTc prolongation was significantly greater compared to MV-TTS (523 ± 52 ms vs. 487 ± 66 ms; *p *= 0.029). Figure 4 depicts two representative examples of the main findings for ECG-1 and ECG-2.

In ECG-3, the previously observed ECG changes tended to regress. The ST-segment showed a clear tendency to return to the isoelectric line in both groups. A small difference between both groups was observed in V3 and V5, with the ST-segment remaining slightly elevated in typical TTS compared to MV-TTS. The T waves also showed a tendency towards normalization, remaining negative in aVL in both groups. The QTc interval shortened in both groups, although it remained longer in typical TTS than in MV-TTS (472 ± 35 ms vs. 423 ± 33 ms; *p* < 0.01).

Supplementary Table S3 summarizes details of R waves analyzed by calculating the difference in R wave magnitude between the different ECGs and showing a significantly greater increase in R waves in leads V4 and V5 from ECG-1 to ECG-2 in typical TTS compared to MV-TTS.

The supplementary material reports data on ST elevation, ST depression, and T wave inversion (Supplementary Table S1) and R wave measurements (Supplementary Table S2) for patients with typical TTS compared to MV-TTS and for each lead in ECG-1, ECG-2, and ECG-3.

## Discussion

This study, conducted in a relatively small yet meticulously characterized population, points to patients with MV-TTS showing distinctive ECG abnormalities. To the best of our knowledge, this is the first study to describe specific patterns affecting the ST-segment, T wave, and QT interval in relation to the time elapsed from symptom onset and involving different leads, probably attributable to a different RWMA distribution.

We found ECG abnormality patterns in MV-TTS compared to typical TTS as follows: (1) on admission, ST-segment depression in inferior leads and V4–V6; (2) at 48 h, differently distributed, less profound and less diffuse T wave inversion; and (3) at 5–7 days, more attenuated QT interval prolongation.

While most of the previous studies have primarily focused on identifying differences between typical TTS and anterior myocardial infarction (AMI) (6–8), only a limited number of studies have explicitly examined electrocardiographic changes in atypical TTS. In general, atypical forms are reported to exhibit less prominent electrocardiographic changes (9). Supplementary Table S4 summarizes the published articles that report any electrocardiographic data comparing typical and atypical TTS (3, 6, 10, 20–27).

In typical TTS form, ST-segment elevation is less pronounced (13, 28) and involves a broader range of leads compared in comparison to anterior AMI (14, 19). Precordial leads from V2 to V6 are primarily affected, and there are no reciprocal changes in the inferior leads (15, 16). Similar electrocardiographic findings were noted in our cohort, where we observed a more substantial ST-segment elevation in leads V2–V5. On the other hand, ST-segment depression is not frequently documented in the literature, even though it is more closely linked to atypical forms (21, 29, 30). In a study published by Ghadri et al. (6), the incidence of ST-segment depression occurs in 7% of typical TTS cases compared to 10.8% in the atypical cases; while Obeid et al. (21) reported 2% incidence of isolated ST-segment depression in typical TTS compared to 10.6% in atypical variants (*p* = 0.019).

In a comparative ECG analysis on admission between typical TTS and MV-TTS that focused on ST-segment elevation and T wave inversion, Kurisu et al. (20) reported that ST-segment elevation was more frequent in MV-TTS in leads V2 and V3, and was not observed in the inferior leads (II, III, aVF) or in V5 or V6. No information regarding ST-segment depression was reported. Those results are aligned with those for our cohort in that ST-segment elevation is more frequently affected and was also greater in leads V2 and V3, although ST-segment elevation occurred in a smaller proportion of our patients with MV-TTS. Additionally, in the leads where ST-segment elevation was not observed in Kurisu's study, we instead observed ST-segment depression.

In our opinion, there are several possible reasons for infrequent reporting of ST-segment depression in MV-TTS. First, most studies analyze typical TTS due to its higher prevalence. Second, in studies that analyze atypical TTS, all subtypes (focal, inverted, midventricular) are included, with differing dyskinesia patterns that likely have distinct ECG manifestations. Third, non-identification of the exact time interval between symptom onset and an ECG may have led to ECG analysis at different timepoints, thus precluding the identification of pattern variations according to the particular time of evolution (i.e., in our series, ST-segment depression was more pronounced in ECGs performed at 12 h than at 48 h from symptom onset). Of note also was the fact that, in the 12-h ECG, very few patients in our cohort showed a negative T wave, a characteristic ECG abnormality in the subacute TTS phase. Four, careful ventriculography analysis is essential to accurately classify the TTS subtype. In our study, we found that a number of patients had been misclassified in regard to the dyskinesia type based on angiogram reports, probably due to a semantic bias in TTS nomenclature (primarily derived from the most common form, the apical dyskinesia).

At 48 h, in both groups we observed ECG evolution, as reported in previous studies, with T wave inversion and QTc interval prolongation (17, 18). Regarding negative T wave differences, we observed less pronounced T wave inversion extent and depth in MV-TTS compared to typical TTS. In MV-TTS, the most negative T waves were observed in I, aVL, and V2, and reached 1 mm at most; in contrast, in typical TTS, negative T waves were observed across limb and precordial leads except for V1, and the most pronounced negative T waves, reaching up to 4 mm, were observed in V3–V5. Similar findings are described in the study by Kurisu et al. (20): T wave inversion in MV-TTS was only present in leads V1–V3, but was more widespread in typical TTS and was particularly marked in the precordial leads from V3 to V6. As for QTc interval prolongation, while observed in both our groups, it was more prominent in typical TTS. This higher prevalence of QTc interval prolongation in typical TTS is possibly explained by its association with T wave inversion (19), more frequently observed in typical TTS.

**Figure 5:**
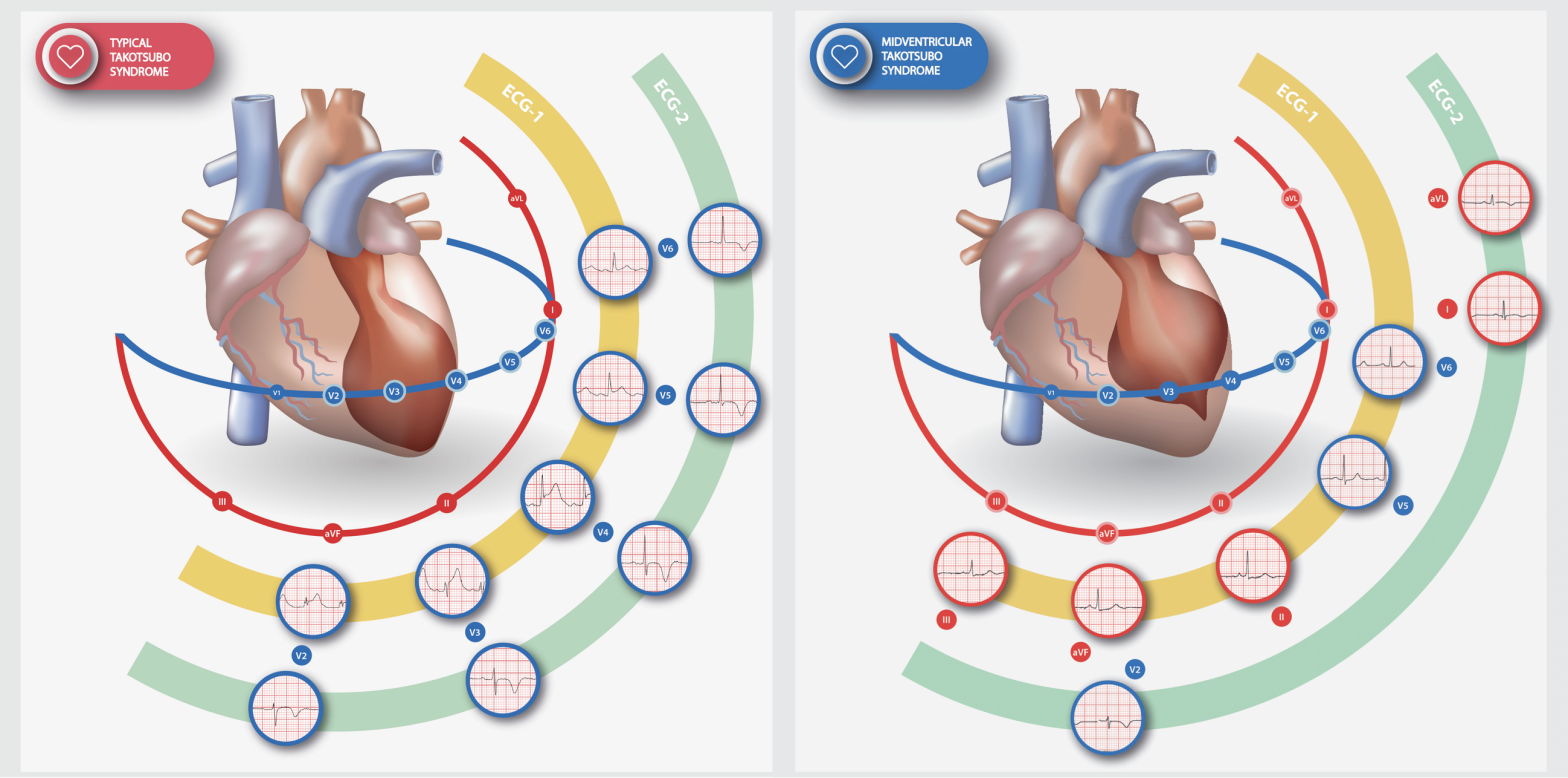
Distinct dyskinesia patterns and their relationship with the most affected ECG leads in typical TTS (left) and MV-TTS (right). ST-segment alterations (elevated in typical TTS, depressed in MV-TTS) and T wave inversion are illustrated for ECG-1 and ECG-2, respectively. Shown is a representative example of the abnormality observed in each lead. ECG, electrocardiogram; ECG-1,12 h from symptom onset; ECG-2, 48 h from symptom onset; TTS, takotsubo syndrome; MV-TTS, midventricular TTS.

The factors contributing to differing ECG patterns in TTS are still not fully understood. Indeed, it seems reasonable to consider that different myocardial dyskinesia patterns would result in different ECG presentations. We observed that the leads that exhibited more pronounced changes could be considered to align with the specific regions affected by each pattern; thus, the anterior precordial leads were more affected in typical TTS, and inferior and lateral leads more in MV-TTS, as illustrated in Figure 5.

Some studies have suggested that a transient reduction in the amplitude of QRS complexes may be indicative of myocardial edema in TTS (31, 32). Additionally, this phenomenon has shown promise potential as an electrocardiographic marker for distinguishing it from acute cardiovascular syndrome (32). In our study, a noteworthy observation was the discrete R wave increase in leads V3–V6 in the 48-h and 7-day ECGs in typical TTS, potentially indicating edema. We believe that such changes may become more apparent over a more extended period of ECG monitoring, especially considering that myocardial edema as detected by cardiac magnetic resonance can persist for over 2 weeks (33–35).

Unresolved questions also remain concerning the pathophysiology underlying TTS (36, 37) and the reasons for diverse patterns of involvement. Consistently, natriuretic peptides levels are higher and LVEF is lower in patients with typical TTS compared to those with atypical TTS (38). This would suggest that typical TTS involves a larger myocardial portion with edema, which, in turn, might contribute to more pronounced ECG changes.

Our study presents various limitations, primarily stemming from the retrospective observational nature of the research. The sample size was limited in both groups, because of meticulous patient selection based on the availability of precisely identified symptom onset times and of ventriculography data. Complete sets of ECGs at all three time points were not available for every patient and clinical follow-up of the patients was not implemented. Finally, our study was a single-center study, with the inherent limitation as to external validation.

## Conclusion

Patients with MV-TTS exhibited a distinctive pattern of ECG abnormalities, marked by ST-segment depression in inferolateral leads in the acute phase, less profound and less extensive T wave inversion that mostly affected leads I, aVL and V2, and attenuated QT interval prolongation, compared to typical TTS.

## Data Availability

The raw data supporting the conclusions of this article will be made available by the authors, without undue reservation.
