# Supplementary material for: Comparative electrocardiographic analysis of midventricular and typical takotsubo syndrome
Source: Front Cardiovasc Med. 2023 Dec 4;10:1286975. doi: 10.3389/fcvm.2023.1286975 (PMC10725917; doi:10.3389/fcvm.2023.1286975)
Supplement: Supplementary file 1 [file Datasheet1.docx]

Supplementary Material

# Supplementary Figures and Tables

# Table S1. Patients with typical TTS and MV-TTS. ST-segment elevation, ST-segment depression, and T wave inversion in each lead in ECG-1, ECG-2, and ECG-3.

|  | **ECG-1** | | **ECG-2** | | **ECG-3** | |
| --- | --- | --- | --- | --- | --- | --- |
|  | **Typical TTS** (n=28) | **MV-TTS**  (n=26) | **Typical TTS**  (n=29) | **MV-TTS**  (n=24) | **Typical TTS**  (n=17) | **MV-TTS**  (n=15) |
| ST-segment elevation, n (%) | | | | | | |
| I | 15 (54) | 4 (15) | 9 (31) | 5 (21) | 2 (12) | 1 (7) |
| II | 16 (57) | 5 (19) | 11 (38) | 2 (8) | 4 (24) | 1 (7) |
| III | 11 (39) | 3 (12) | 7 (24) | 2 (8) | 4 (24) | 2 (13) |
| aVR | 4 (14) | 5 (19) | 8 (28) | 4 (17) | 2 (12) | 2 (13) |
| aVL | 6 (21) | 8 (31) | 5 (17) | 2 (8) | 0 | 3 (20) |
| aVF | 11 (39) | 3 (12) | 9 (31) | 4 (17) | 2 (12) | 1 (7) |
| V1 | 7 (25) | 0 | 6 (21) | 4 (17) | 3 (18) | 2 (13) |
| V2 | 17 (61) | 12 (46) | 17 (59) | 3 (13) | 6 (35) | 1 (7) |
| V3 | 19 (68) | 8 (31) | 14 (48) | 3 (13) | 5 (29) | 2 (13) |
| V4 | 17 (61) | 4 (15) | 13 (45) | 1 (4) | 5 (29) | 1 (7) |
| V5 | 16 (57) | 1 (4) | 12 (41) | 2 (8) | 3 (18) | 0 |
| V6 | 11 (39) | 1 (4) | 7 (24) | 2 (8) | 2 (12) | 1 (7) |
| ST-segment depression, n (%) | | | | | | |
| I | 1 (4) | 5 (19) | 0 | 5 (21) | 2 (12) | 2 (13) |
| II | 2 (7) | 13 (50) | 2 (7) | 7 (29) | 2 (12) | 0 |
| III | 1 (4) | 14 (54) | 3 (10) | 5 (21) | 1 (6) | 1 (7) |
| aVR | 3 (11) | 11 (42) | 0 | 4 (17) | 3 (18) | 0 |
| aVL | 1 (4) | 3 (12) | 2 (7) | 3 (13) | 1 (6) | 0 |
| aVF | 2 (7) | 14 (54) | 4 (14) | 6 (25) | 2 (12) | 1 (7) |
| V1 | 1 (4) | 4 (15) | 1 (3) | 1 (4) | 0 | 0 |
| V2 | 0 | 2 (8) | 0 | 4 (17) | 0 | 0 |
| V3 | 2 (7) | 6 (23) | 1 (4) | 6 (26) | 1 (6) | 2 (13) |
| V4 | 1 (4) | 12 (46) | 4 (14) | 8 (33) | 1 (6) | 1 (7) |
| V5 | 1 (4) | 10 (38) | 1 (3) | 6 (25) | 1 (6) | 2 (13) |
| V6 | 1 (4) | 6 (23) | 2 (7) | 5 (21) | 1 (6) | 1 (7) |
| T wave inversion, n (%) | | | | | | |
| I | 0 | 2 (8) | 20 (69) | 12 (50) | 4 (24) | 5 (33) |
| II | 1 (4) | 0 | 21 (72) | 7 (29) | 4 (24) | 3 (20) |
| III | 2 (7) | 4 (15) | 14 (48) | 7 (29) | 1 (6) | 2 (13) |
| aVR | 2 (7) | 2 (7) | 22 (76) | 10 (42) | 5 (29) | 4 (27) |
| aVL | 9 (32) | 13 (50) | 12 (41) | 14 (58) | 6 (35) | 5 (33) |
| aVF | 2 (7) | 1 (4) | 19 (66) | 6 (25) | 2 (12) | 2 (13) |
| V1 | 10 (36) | 13 (50) | 5 (17) | 9 (38) | 2 (12) | 4 (27) |
| V2 | 1 (4) | 4 (15) | 15 (52) | 14 (58) | 3 (18) | 5 (33) |
| V3 | 1 (4) | 1 (4) | 26 (90) | 13 (54) | 5 (29) | 4 (27) |
| V4 | 3 (11) | 1 (4) | 27 (93) | 9 (38) | 6 (35) | 6 (40) |
| V5 | 3 (11) | 1 (4) | 27 (93) | 9 (38) | 8 (47) | 7 (47) |
| V6 | 2 (7) | 0 | 26 (90) | 9 (38) | 6 (35) | 4 (27) |

ST-segment elevation was considered present if ≥0.5 mm in limb leads and ≥1 mm in precordial leads. ST-segment depression was considered present if ≥0.5 mm in any lead. T wave inversion was considered present if ≥1 mm in any lead. ST-segment elevation, ST-segment depression, and T wave inversion, when present, were measured to the nearest 0.5 mm in all leads.

ECG, electrocardiogram; ECG-1, 12 hours from symptom onset; ECG-2, 48 hours from symptom onset; ECG-3, 5-7 days from symptom onset; TTS, takotsubo syndrome; MV-TTS, midventricular TTS.

**Table S2.** Patients with typical TTS and MV-TTS. R wave (mm) in each lead in ECG-1, ECG-2, and ECG-3.

**a**. **ECG-1**

|  | **Typical TTS**  (n=28) | **MV-TTS**  (n=26) | **p value** |
| --- | --- | --- | --- |
| I | 5.2 [4.15 – 7] | 4.6 [3.8 – 7.6] | 0.869 |
| II | 3.3 [1.6 – 5.4] | 6.2 [3.3 – 8.7] | **0.003** |
| III | 0.75 [0.35 – 3] | 1.7 [0.8 – 4.3] | 0.036 |
| aVR | 3.7 [2.6 – 5.1] | 5 [3.6 – 6.8] | **0.040** |
| aVL | 4.1 [2.6 – 6.4] | 3.6 [1 – 5.3] | 0.272 |
| aVF | 1.55 [0.3 – 3.4] | 3.6 [1.8 – 6.6] | **0.003** |
| V1 | 0.95 [0.6 – 2] | 0.65 [0.1 – 1.3] | **0.049** |
| V2 | 0.95 [0.2 – 2.1] | 0.4 [0.2 – 1.6] | 0.466 |
| V3 | 1.15 [0.4 – 3.9] | 2.8 [0.6 – 4.4] | 0.286 |
| V4 | 3.35 [1.4 – 8.1] | 7.4 [4.7 – 11.8] | **0.007** |
| V5 | 4.3 [3.05 – 6.9] | 9.75 [6.6 – 13] | **< 0.001** |
| V6 | 4.25 [2-85 – 6.8] | 8.1 [5.1 – 10.3] | **< 0.001** |

**b**. **ECG-2**

|  | **Typical TTS**  (n=29) | **MV-TTS**  (n=24) | **p value** |
| --- | --- | --- | --- |
| I | 5.3 [4.1 – 7.7] | 5.55 [3.2 – 7.3] | 0.782 |
| II | 4 [2 – 4.3] | 5.15 [3.7 – 6.4] | **0.022** |
| III | 0.7 [0.3 – 1.8] | 1.1 [0.6 – 3.4] | 0.135 |
| aVR | 4.1 [3.3 – 5.2] | 4.9 [3.4 – 6.3] | 0.183 |
| aVL | 4.4 [2.4 – 6.4] | 3.3 [1.7 – 5.2] | 0.161 |
| aVF | 1.4 [0.3 – 2.6] | 2.7 [1.7 – 4] | **0.010** |
| V1 | 0.8 [0.6 – 1.3] | 0.95 [0.2 – 1.6] | 0.830 |
| V2 | 1.2 [0.6 – 2.3] | 1.05 [0.6 – 3.1] | 0.950 |
| V3 | 3.4 [1.6 – 6] | 3.2 [0.9 – 6.7] | 0.964 |
| V4 | 7.7 [4.7 – 9.2] | 7.7 [4.5 – 10.5] | 0.475 |
| V5 | 5.9 [4.8 – 8.7] | 8.7 [6.5 – 10.9] | 0.075 |
| V6 | 5.1 [4 – 6.3] | 7.75 [5.7 – 9.3] | **0.008** |

**c. ECG-3**

|  | **Typical TTS**  (n=17) | **MV-TTS**  (n=15) | p value |
| --- | --- | --- | --- |
| I | 4.9 [3.8 – 7.8] | 6.7 [3.1 – 9.5] | 0.664 |
| II | 4 [2.4 – 5.5] | 6.2 [5.1 – 8.6] | **0.013** |
| III | 1.5 [0.5 – 2.2] | 1.6 [0.7 – 3] | 0.298 |
| aVR | 4.4 [3.1 – 5.9] | 5.2 [4.5 – 8] | 0.067 |
| aVL | 3.4 [2.7 – 6.4] | 5.2 [2 – 6.9] | 0.763 |
| aVF | 1.7 [0.2 – 3.3] | 3.4 [1.1 – 5.2] | 0.113 |
| V1 | 0.8 [0.5 – 1] | 0.9 [0.1 – 1.4] | 0.925 |
| V2 | 1.6 [0.9 – 2.7] | 1.6 [0.7 – 3.1] | 0.895 |
| V3 | 4.3 [0.8 – 8.4] | 4.9 [3.5 – 6.5] | 0.438 |
| V4 | 6.9 [2.8 – 9.2] | 8.5 [6.8 – 12.5] | 0.193 |
| V5 | 7.1 [4.9 – 8.6] | 9.1 [7.1 – 11.8] | 0.057 |
| V6 | 6.2 [4.8 – 7.3] | 7.6 [6.1 – 10.1] | **0.033** |

Data are expressed as median [interquartile range].

ECG, electrocardiogram; ECG-1,12 hours from symptom onset; ECG-2, 48 hours from symptom onset; ECG-3, 5-7 days from symptom onset; TTS, takotsubo syndrome; MV-TTS, midventricular TTS.

|  | A (ECG2 - ECG1) | | | B (ECG3 - ECG2) | | |
| --- | --- | --- | --- | --- | --- | --- |
|  | **Typical TTS**  (n = 24) | **MV-TTS**  (n = 23) | **p value** | **Typical TTS**  (n = 16) | **MV-TTS**  (n = 15) | **p value** |
| I | 0 [-1.4 – 0.9] | -0.3 [-1.5 – 1.5] | ns | 0.3 [-0.2 – 0.95] | 0.2 [-0.5 – 1.3] | ns |
| II | 0.1 [-0.45 – 0.85] | -0.9 [-1.8 – 0.5] | ns | 0.2 [-0.5 – 0.75] | 1 [0.5 – 1.4] | 0.028 |
| III | 0 [-0.5 – 0.3] | -0.1 [-1.2 – 0.8] | ns | 0.15 [-0.15 – 0.55] | 0.5 [-0.5 – 1.3] | ns |
| aVR | 0.35 [-0.75 – 0.85] | 0 [-1.2 – 1.3] | ns | 0.7 [-0.1 – 1.15] | 0.3 [-0.2 – 1.4] | ns |
| aVL | -0.25 [-1.05 – 0.85] | 0 [-1.4 – 1.1] | ns | 0.05 [-0.35 – 0.65] | -0.4 [-0.9 – 2.3] | ns |
| aVF | 0 [-0.65 – 0.2] | -0.5 [-1.7 – 0.8] | ns | 0.15 [-0.4 – 1.25] | 0.5 [-0.7 – 1.6] | ns |
| V1 | -0.05 [-0.45 – 0.2] | 0 [-0.3 – 0.5] | ns | -0.05 [-0.45 – 0.1] | 0 [0 – 0.1] | ns |
| V2 | 0.1 [-0.3 – 0.7] | 0.4 [-0.1 – 1.2] | ns | 0 [-0.15 – 1.05] | 0.1 [-0.3 – 0.9] | ns |
| V3 | 0.75 [0.05 – 2.65] | 0.3 [-0.7 – 2.6] | ns | 0.45 [-0.2 – 2.5] | 0.7 [0.1 – 3.2] | ns |
| V4 | 2 [0.45 – 5.1] | -0.8 [-3.5 – 4.6] | 0.016 | 0.25 [-1.35 – 2.2] | 3 [0.8 – 4.8] | ns |
| V5 | 1.75 [0.1 – 3.75] | -1.5 [-3 – 1.7] | 0.001 | 0.65 [-1.2 – 1.4] | 1.5 [0.3 – 3.2] | ns |
| V6 | 1.25 [-0.05 – 1.7] | -0.4 [-1.7 – 1.5] | ns | 0.8 [0 – 1.8] | 0.7 [-2 – 2.2] | ns |

**Table S3**. R wave differences (in mm) for each lead, calculated by subtracting amplitudes from ECG-1 to ECG-2 (A) and from ECG-2 to ECG-3 (B).

Data are expressed as median [interquartile range].

ECG, electrocardiogram; ECG-1,12 hours from symptom onset; ECG-2, 48 hours from symptom onset; ECG-3, 5-7 days from symptom onset; ns, non-significant; TTS, takotsubo syndrome; MV-TTS, midventricular TTS.

| **Reference** | **Population/sample** | **ST-segment elevation** | **ST-segment depression** | **T wave inversion** |
| --- | --- | --- | --- | --- |
| Kurisu et al. Clin. Cardiol 2011^20^ # | 20 typical TTS vs 6 MV-TTS | Sum of ST-segment elevation in leads V4 to V6: 3.5+-3.0 mm typical vs 0.4+-0.8 mm MV (p<0.05). | Not reported | Typical: more common leads are V3-V6 (>50%).  MV: T wave inversion only observed in V1-V3 (30-40%). |
| Obeid S et al. A929 JACC 2017 ^21^ # | 200 patients total | Anterior ST elevation: 28% typical vs 4% atypical  (p<0.001)  Antero-septal ST elevation: 42% typical vs 19% atypical (p=0.005) | Isolated ST depression: 2% typical vs 10.6% atypical (p=0.019 | Inferior T wave inversion: 20% typical vs 2% atypical  (p=0.002) |
| Ghadri et al. JAMA Cardiol 2016 ^6^ | 1430 typical TTS vs 320 atypical TTS (MV, basal, and focal) | 45.9% typical vs 33.9% atypical  (p<0.001) | 7% typical vs 10.8% atypical  (p=0.03) | 42.4% typical vs 35% atypical  (p=0.02) |
| Kato et al. Circulation Journal 2016 ^22^ | 85 typical TTS vs 49 MV-TTS vs 10 focal | 61 (72%) typical vs 37 (76%) MV vs 4 (40%) focal  (p=0.10) | Not reported | 53 (62%) typical vs 31 (65%) MV vs 7 (70%) focal  (p=0.89) |
| Gaede et al IJC Heart & Vasculature 2021 ^10^ | 89 typical TTS vs 37 atypical TTS (MV, basal, and focal) | 20% typical vs 16% atypical | 2% typical vs 8% atypical | 48% typical vs 27% atypical |
| Cheng-Kang Chen et al. Acta Cardiol Sin 2013 ^23^ | 14 typical TTS vs 6 atypical TTS (MV, basal, and focal) | 12 (86%) typical vs 2 (33%) atypical | Not reported | Not reported |
| Kwon S et al. International Journal of Cardiology 2013 ^24^ | 140 typical TTS vs 68 atypical TTS (MV, basal, and focal) | 45 (32.1%) typical vs 9 (13.2%) atypical (p 0.004) | Not reported | 93 (66.4%) typical vs 35% (51.5%) atypical (p 0.038) |
| Haghi et al. International Journal of Cardiology 2007 ^25^ | 10 typical TTS vs 7 atypical TTS (MV, basal, and focal) | 6 typical vs 2 atypical | 0 atypical vs 4 atypical | 10 typical vs 4 atypical |
| Murthy et al. Cardiol Res. 2014 ^26^ | 7 typical TTS vs 4 atypical TTS (MV, basal, and focal) | 2 typical vs 0 atypical | 0 | 4 typical vs 2 atypical |
| Rellini et al. J Cardiovasc Med (Hagerstown) 2018 ^27^ | 74 typical TTS vs 30 atypical TTS (MV, basal, and focal) | 43 (58%) typical vs 8 (27%) atypical | 3 (4%) typical vs 4 (13%) atypical | 22 (30%) typical vs 8 (27%) atypical |
| Kurowski et al. Chest 2007 ^3^ | 22 typical TTS vs 13 MV-TTS | 63.6% typical vs 76.9% MV (p 0.41) | Not reported | Not reported |

**Table S4.** Published studies reporting data on ECG abnormalities in different TTS variants.

# The main objective was to compare ECG differences between groups. The remaining studies report ECG changes within the clinical characteristics of the patients.

ECG, electrocardiogram; MV, midventricular; TTS, takotsubo syndrome; MV-TTS, midventricular TTS.
